# Clinical Significance of the Edema Index in Incident Peritoneal Dialysis Patients

**DOI:** 10.1371/journal.pone.0147070

**Published:** 2016-01-19

**Authors:** Seok Hui Kang, Eun Woo Choi, Jong Won Park, Kyu Hyang Cho, Jun Young Do

**Affiliations:** Division of Nephrology, Department of Internal Medicine, Yeungnam University Hospital, Daegu, Republic of Korea; Sao Paulo State University, BRAZIL

## Abstract

**Background:**

Proper monitoring for volume overload is important to improve prognosis in peritoneal dialysis (PD) patients. The association between volume status and residual renal function (RRF) remains an unresolved issue. The aim of the present study was to evaluate the association between the edema index and survival or RRF in incident PD patients.

**Patients and Methods:**

We identified all adults who underwent PD. The edema index was defined as the ratio of extracellular fluid to total body fluid. Participants with available data regarding survivorship or non-survivorship during the first year after PD initiation were included in the area under the receiver operating characteristic curve analysis. The cutoff value of the edema index for 1-year mortality was >0.371 in men and >0.372 in women. Participants were divided into two groups according to the cutoff value of their baseline edema indices: High (>cutoff value) and Low (≤cutoff value). Survivors during the first year after PD initiation were divided into two groups according to the initial and 1-year edema index: Non-improvement (maintenance of criteria in the initial Low group during the year) and Other (all participants except those in the Non-improvement group).

**Results:**

In total, 631 patients were enrolled in the present study. The cutoff value of the edema index for 1-year mortality was >0.371 in men and >0.372 in women. The respective mean initial RRF values (mL·min^-1^·1.73 m^-2^) in the Low and High groups, respectively, were 4.88 ± 4.09 and 4.21 ± 3.28 in men (*P* = 0.108), and 3.19 ± 2.57 and 2.98 ± 2.70 in women (*P* = 0.531). There were no significant differences between groups in either sex. The respective mean RRF values at 1 year after PD initiation in the Low and High groups, respectively, were 3.56 ± 4.35 and 2.73 ± 2.53 in men, and 2.80 ± 2.36 and 1.85 ± 1.51 in women. RRF at 1 year after PD initiation was higher in the Low group than in the High group (men: *P* = 0.027; women: *P* = 0.001). In men, the cumulative 5-year survival rates were 78.7% and 46.2% in the Low and High groups, respectively, whereas in women, rates were 77.2% and 58.8% in the Low and High groups, respectively. For survivors during the first year after PD initiation, the Non-improvement group was associated with a poor survival rate compared with the Other group for both sexes.

**Conclusion:**

A high edema index was associated with mortality in incident PD patients at baseline and follow-up. The edema index may be used as a new marker for predicting mortality in PD patients.

## Introduction

Peritoneal dialysis (PD) is one of the renal replacement therapies that patients with end-stage renal disease commonly undergo [1]. Volume status is an important risk factor for morbidity and mortality in PD patients [2–5]. Volume overload is associated with hypertension, malnutrition, inflammation, and endothelial dysfunction, which induces major cardiovascular complications such as left ventricular hypertrophy. Consequently, previous studies have shown that volume overload is associated with higher mortality in PD patients [2–5]. Therefore, monitoring and proper intervention for volume overload are important to improve survival in PD patients.

Maintenance of residual renal function (RRF) is important to improve prognosis in PD patients. It is known that PD leads to better preservation of RRF compared with hemodialysis [6]. However, the association between volume status and RRF remains an unresolved issue in PD patients. A decrease in RRF is associated with volume overload; however, whether overhydration or proper hydration status influence a change in RRF is not clear. Further studies examining hydration status and RRF are needed to identify the association between the two variables.

Volume status can be evaluated by vena cava-diameter using ultrasonography or chest radiography, in addition to biomarkers, clinical history, or physical examination [7–11]. However, there are no current guidelines available for evaluating volume status. A dilution method using deuterium oxide or bromide is the gold standard for predicting volume status [12]. However, this method requires expensive equipment and a relatively long evaluation period, and evaluation in an outpatient setting using this method is difficult. Multi-frequency bioimpedance analysis (BIA) is a simple and accurate body composition analyzer. BIA applies alternating currents to the body and obtains eight-polar tactile-electrode impedances [13,14]. These impedances are obtained using differences in cell membrane permeability according to variable frequencies. There have been improvements in the BIA technique, in particular in term of its accuracy. Extracellular fluid (ECF) and total body fluid (TBF) are calculated using these impedance measurements. Chamney et al. showed that pre-hemodialysis patients have a higher slope for interdialytic weight gain than a normovolemic population, and this increases to a greater extent in ECF than in TBF [15].

Regarding differences in body size and hypervolemic status, the edema index as an independent indicator is obtained by calculating the ECF/TBF ratio. Previous studies have demonstrated an association between volume status measured by dilution methods and the edema index [13,14]. Therefore, the edema index measured by BIA may be a useful marker for defining volume status, and it may be associated with mortality in dialysis patients. However, there are few studies evaluating the clinical impact of the edema index in PD patients. The aim of the present study was to evaluate the association between the edema index and survival or RRF in incident PD patients.

## Methods

### Study population

We reviewed medical records at the Yeungnam University Hospital in Korea and identified all adults (> 18 years) who underwent PD between January 2001 and April 2014 (S1 Fig). Sixty-three participants refused to be subjected to baseline BIA measurements. Cardiac defibrillators and pacemakers are contraindications for BIA measurements, but there were no participants with these devices in the present study. The participants in the present study were divided into 2 cohorts: the Total cohort and the FU cohort. The Total cohort included all incident PD patients with baseline BIA measurements. The FU cohort included incident PD patients with BIA measurements at both baseline and 1 year after initiation of PD. This study was approved by the institutional review board of Yeungnam University Hospital (2015-07-040). The board waived the need for informed consent, as subjects’ records and information were anonymized and de-identified before the analysis.

### Clinical information

Clinical and laboratory data collected 1 month after the initiation of PD included age, sex, the Davies risk index, dialysis modality, body mass index (BMI; kg/m^2^), RRF (mL·min^-1^·1.73 m^-2^), serum albumin level (g/L), C-reactive protein level (CRP; mg/dL), dialysate/plasma creatinine ratio (D/P Cr), weekly Kt/Vurea, and the edema index.

Comorbidities were graded according to the Davies risk index, and included ischemic heart disease, peripheral vascular disease, left ventricular dysfunction, diabetes mellitus, systemic collagen vascular disease, and other significant pathologies [16]. Co-morbidities were graded as low risk (0), intermediate risk (1–2), or high risk (≥3). BMI was calculated by dividing the total body weight in kilograms by the square of the patient’s height in meters (kg/m²). RRF was calculated using 24-hour urine results derived from the mean urea clearance and creatinine clearance values corrected for a body surface area of 1.73 m^2^. Serum albumin and CRP levels were measured with an Olympus AU4500 automatic chemical analyzer (Olympus, Tokyo, Japan); bromocresol green was used to detect albumin.

At our center, body composition analysis by BIA is routinely recommended annually for PD patients after obtaining their informed consent. BIA was determined with the Inbody 4.0 Body Composition Analyzer (Biospace, Seoul, Korea) using multi-frequency and segmental analyses. TBF and ECF values were obtained from the BIA. The edema index was defined as the ratio of ECF to TBF. BIA was performed on an “empty” abdomen (no dialysate in the peritoneal cavity). For the Total cohort, participants were divided into two groups according to the cutoff value of their baseline edema index: High (>cutoff value) and Low (≤cutoff value). For the FU cohort, participants were divided into two groups according to the initial and 1-year edema index: Non-improvement (maintenance of criteria in the initial Low group during the year) and Other (all participants except those in the Non-improvement group).

A modified 4.25% peritoneal equilibration test (PET) was performed. The intra-abdominal fluid was drained, and the PD fluid containing 4.25% glucose was infused intraperitoneally. The creatinine level of the drained dialysate 4 hours after the injection was divided by the blood creatinine level to obtain the D/P Cr. A high transporter was defined when D/P Cr was >0.81. Weekly dialysis dose, Kt/V urea, was calculated based on 24-hour urine and dialysate as follows: weekly Kt/V urea = 7 × {[24-hour urine urea nitrogen content (mg/dL) × 24-hour urine volume (L)] + [24-hour dialysate urea nitrogen content (mg/dL) × 24-hour drain volume (L)]} ÷ [distribution volume of urea (L) × serum urea nitrogen (mg/dL)]. The distribution volume of urea was calculated using Watson’s formula [17]. The RRF was calculated based on 24-h urine collection as follows: RRF = 0.5 × urine volume × [24-h urine creatinine concentration (mg/dL)/serum creatinine (mg/dL) + 24-h urine urea nitrogen concentration (mg/dL)/serum urea nitrogen (mg/dL)] × 1.73/body surface area (m^2^) [18].

Ultrafiltration failure (UFF) was defined as a volume <400 mL of the net ultrafiltration in a 4-h dwell using 2 L of 4.25% dialysate [19]. Type I, II, and III UFF was defined as 0.81<D/P Cr, D/P Cr<0.5, and 0.5<D/P Cr<0.81, respectively.

### Statistical analyses

Data were analyzed using SPSS, version 21 (SPSS, Chicago, IL, USA). Categorical variables were expressed as numbers and percentages. Continuous variables were expressed as mean ± standard deviation. The Pearson χ^2^ test or Fisher exact test was used to analyze categorical variables. For continuous variables, the means were compared using the Student *t*-test or multivariate analysis of variance. Discrimination, which is the model’s ability to differentiate between patients who exhibited survivorship or non-survivorship during the first year after PD initiation, was examined using the area under the receiver operating characteristic curve (AUROC). AUROC analysis was also performed to calculate cutoff values, sensitivity, and specificity. The cutoff risk point was defined as the highest sensitivity (1-specificity) value in the AUROC. The AUROC was calculated using MedCalc, version 11.6.1.0 (MedCalc, Mariakerke, Belgium). Multivariate analysis for RRF was performed using analysis of covariance and was adjusted for age, initial RRF, initial CRP, Davies risk index, BMI, and presence of high peritoneal transport status. The survival estimates were calculated using Kaplan–Meier and Cox regression analyses. Multivariate Cox regression analyses were adjusted for age, initial CRP, initial RRF, Davies risk index, and BMI, and evaluated using the multivariate model by the enter method. In this model, covariates are selected by possible association with mortality in PD patients. Assumptions of univariate and multivariate Cox regression analyses were tested, and cumulative incident plots demonstrate congruent inflection. *P*-values for the comparison of survival curves were determined by the log-rank test. The level of statistical significance was set at *P* < 0.05.

## Results

### Clinical characteristics of the participants

The Total cohort consisted of 631 patients who were enrolled in the present study (Table 1). The proportion of men was higher (n = 341, 54.0%). Mean age at the start of PD was 54.1 ± 13.7 years in men and 52.7 ± 14.2 years in women (*P* = 0.219). There were no significant differences in BMI, CRP, or serum albumin levels between men and women. The proportion of participants who underwent automated PD (APD) and had a high Davies risk index, which was greater in men than in women. Moreover, the RRF was higher in men than in women, and the Kt/V urea in the low-, intermediate, and high Davies risk groups were 2.11 ± 0.72, 2.32 ± 0.77, and 2.21 ± 0.77, respectively (*P* = 0.085).

**Table 1:** Baseline characteristics at peritoneal dialysis initiation.

	Total (n = 631)	Men (n = 341)	Women (n = 290)	*P*-value
Age (years)	53.4 ± 13.9	54.1 ± 13.7	52.7 ± 14.2	0.219
Body mass index (kg/m^2^)	23.5 ± 3.3	23.6 ± 2.0	23.5 ± 3.7	0.587
RRF (mL⋅min^-1^⋅1.73 m^-2^)	3.91 ± 3.37	4.60 ± 3.78	3.11 ± 2.61	<0.001
C-reactive protein (mg/dL)	0.70 ± 1.65	0.67 ± 1.37	0.74 ± 1.93	0.617
Serum albumin (g/L)	34.5 ± 5.6	34.4 ± 6.0	34.7 ± 5.0	0.401
Peritoneal transport characteristics				0.025
High transporter	77 (12.2%)	42 (12.3%)	35 (12.1%)	
High average transporter	342 (54.2%)	202 (59.2%)	140 (48.3%)	
Low average transporter	175 (27.7%)	80 (23.5%)	95 (32.8%)	
Low transporter	37 (5.9%)	17 (5.0%)	20 (6.9%)	
Weekly Kt/Vurea	2.34 ± 1.39	2.25 ± 0.76	2.45 ± 1.87	0.095
PD modality (APD)	100 (15.8%)	71 (20.8%)	29 (10.0%)	<0.001
Davies risk index				0.021
Low	221 (35.0%)	105 (30.8%)	116 (40.0%)	
Intermediate	375 (59.4%)	212 (62.2%)	163 (56.2%)	
High	35 (5.5%)	24 (7.0%)	11 (3.8%)	

Data are expressed as numbers (percentages) for categorical variables and means ± standard deviations for continuous variables. The *P* values were tested with t-test for continuous variables, and the Pearson χ^2^ test or Fisher exact test was used to analyze categorical variables.

Abbreviations: RRF, residual renal function; PD, peritoneal dialysis; APD, automated peritoneal dialysis.

The low transporter participants included 17 men and 20 women. Among low transporters, 5 (29.4%) men underwent APD, and all women low transporters underwent continuous ambulatory PD (CAPD). The edema index value in participants who underwent CAPD and APD was 0.369 ± 0.031 and 0.362 ± 0.025, respectively (*P* = 0.026). For the CAPD and APD patient groups, the respective initial CRP level was 0.72 ± 1.74 mg/dL and 0.63 ± 1.08 mg/dL (*P* = 0.618); initial RRF was 3.82 ± 3.21 mL⋅min^-1^⋅1.73 m^-2^ and 4.41 ± 4.09 mL⋅min^-1^⋅1.73 m^-2^ (*P* = 0.117), and initial serum albumin level was 34.3 ± 5.6 g/L and 35.8 ± 5.2 g/L (*P* = 0.014). Finally, a high Davies risk index was observed in 30 (5.6%) and 5 (5.0%), of participants who underwent CAPD and APD, respectively (*P* = 0.190).

Of the 77 high transporters, 27 (35.1%) had type I UFF and 4 (10.8%) of the 37 low transporters had type II UFF. Of the 517 low or high average transporters, 59 (11.4%) had type III UFF. Of the high transporters, 18.2% and 15.7% of the others underwent APD (*P* = 0.583). Use of icodextrin was 55.8% in high transporters and 15.7% in others (*P* < 0.001). There was no significant difference in use of APD between high transporters and others, but use of icodextrin was higher in high transporters than in others.

### Area under the receiver operating characteristic curve analyses of the edema index

**Fig 1:**
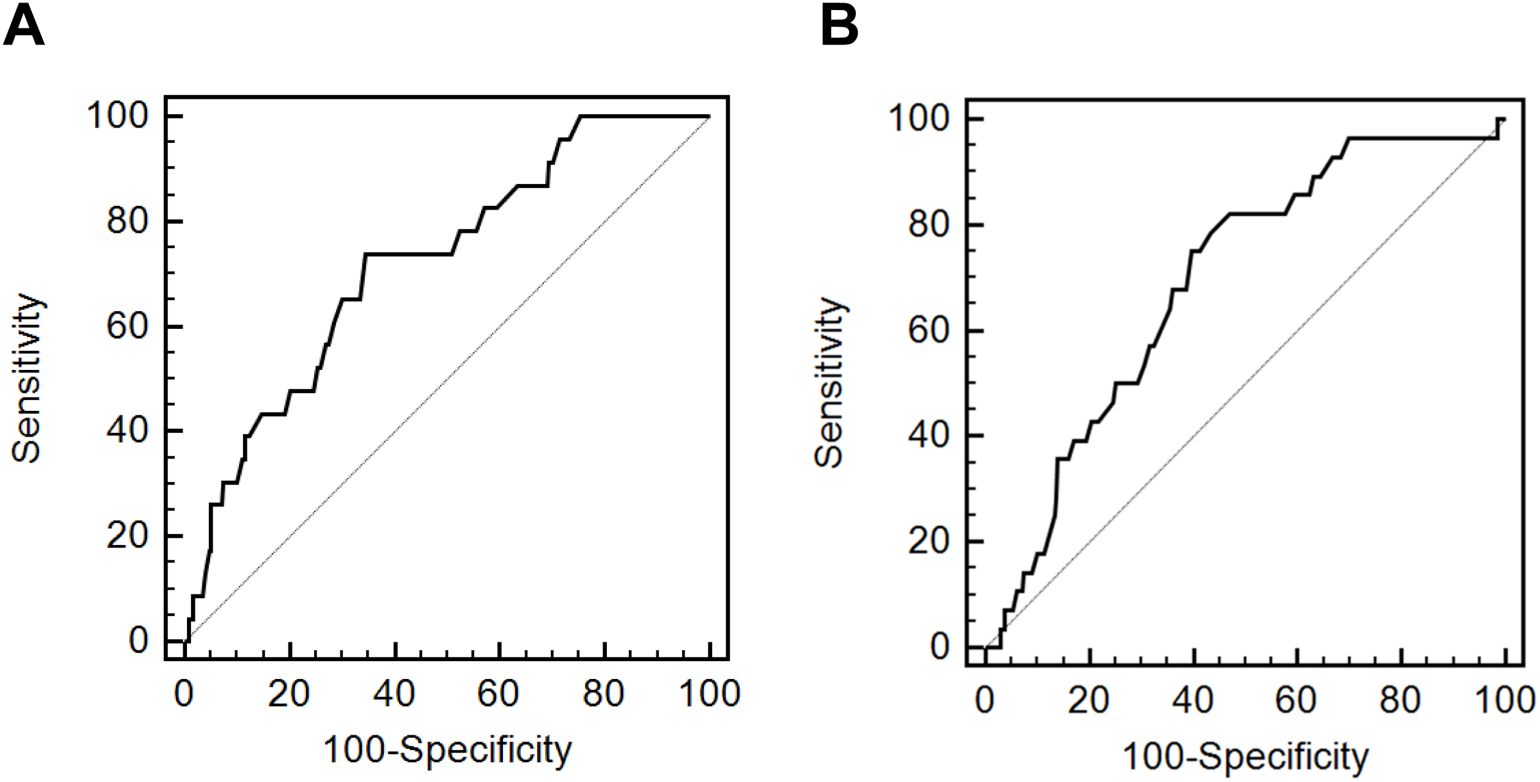
Receiver operating characteristic curves of the edema index for 1 year mortality rates. A. Men. B. Women.

For both sexes, participants with data regarding survivorship or non-survivorship during the first year of follow-up after PD initiation were included in the AUROC analysis. In the male group, 252 survivors and 23 non-survivors were included, while the female group included 293 survivors and 28 non-survivors. The AUROC analysis is shown in Fig 1. The cutoff value of the edema index for 1-year mortality was >0.371 in men and >0.372 in women. AUCs of the edema index values were 0.720 (range, 0.663–0.772) in men and 0.691 (range, 0.637–0.741) in women. In men, the sensitivity and specificity for predicting 1-year mortality were 73.9% and 65.5%, respectively, and in women, 75.0% and 60.4%, respectively.

### Clinical characteristics according to the initial edema index

Patients in the High group were older than those in the Low group of either sex (Table 2). The serum albumin level was lower in the High group than in the Low group, and the intermediate Davies risk index was greater in the High group than in the Low group of either sex. There were no significant differences in the weekly Kt/V urea, BMI, and CRP level of either sex.

**Table 2:** Clinical characteristics according to the initial edema index.

	Men			Women		
	Low (n = 190)	High (n = 151)	*P*-value	Low (n = 181)	High (n = 109)	*P*-value
Age (years)	50.9 ± 14.1	58.1 ± 12.0	<0.001	50.1 ± 13.4	57.0 ± 14.6	<0.001
Body mass index (kg/m^2^)	23.7 ± 2.9	23.5 ± 3.1	0.528	23.4 ± 3.5	23.6 ± 4.0	0.714
C-reactive protein (mg/dL)	0.61 ± 1.33	0.76 ± 1.42	0.332	0.76 ± 2.18	0.71 ± 1.42	0.813
Serum albumin (g/L)	36.9 ± 5.2	31.2 ± 5.4	<0.001	36.3 ± 4.7	32.1 ± 4.5	<0.001
High transporter	19 (10.0%)	23 (15.2%)	0.076	16 (8.8%)	19 (17.4%)	0.012
Weekly Kt/Vurea	2.30 ± 0.80	2.17 ± 0.69	0.110	2.42 ± 2.28	2.51 ± 0.66	0.638
PD modality (APD)	48 (25.3%)	23 (15.2%)	0.023	16 (8.8%)	13 (11.9%)	0.381
Davies risk index			<0.001			<0.001
Low	84 (44.2%)	21 (13.9%)		91 (50.3%)	25 (22.9%)	
Intermediate	99 (52.1%)	113 (74.8%)		84 (46.4%)	79 (72.5%)	
High	7 (3.7%)	17 (11.3%)		6 (3.3%)	5 (4.6%)	

Data are expressed as numbers (percentages) for categorical variables and means ± standard deviations for continuous variables. The *P* values were tested with t-test for continuous variables and the Pearson χ^2^ test or Fisher exact test was used to analyze categorical variables.

Abbreviations: PD, peritoneal dialysis; APD, automated peritoneal dialysis.

### Change in residual renal function according to initial edema index

**Fig 2:**
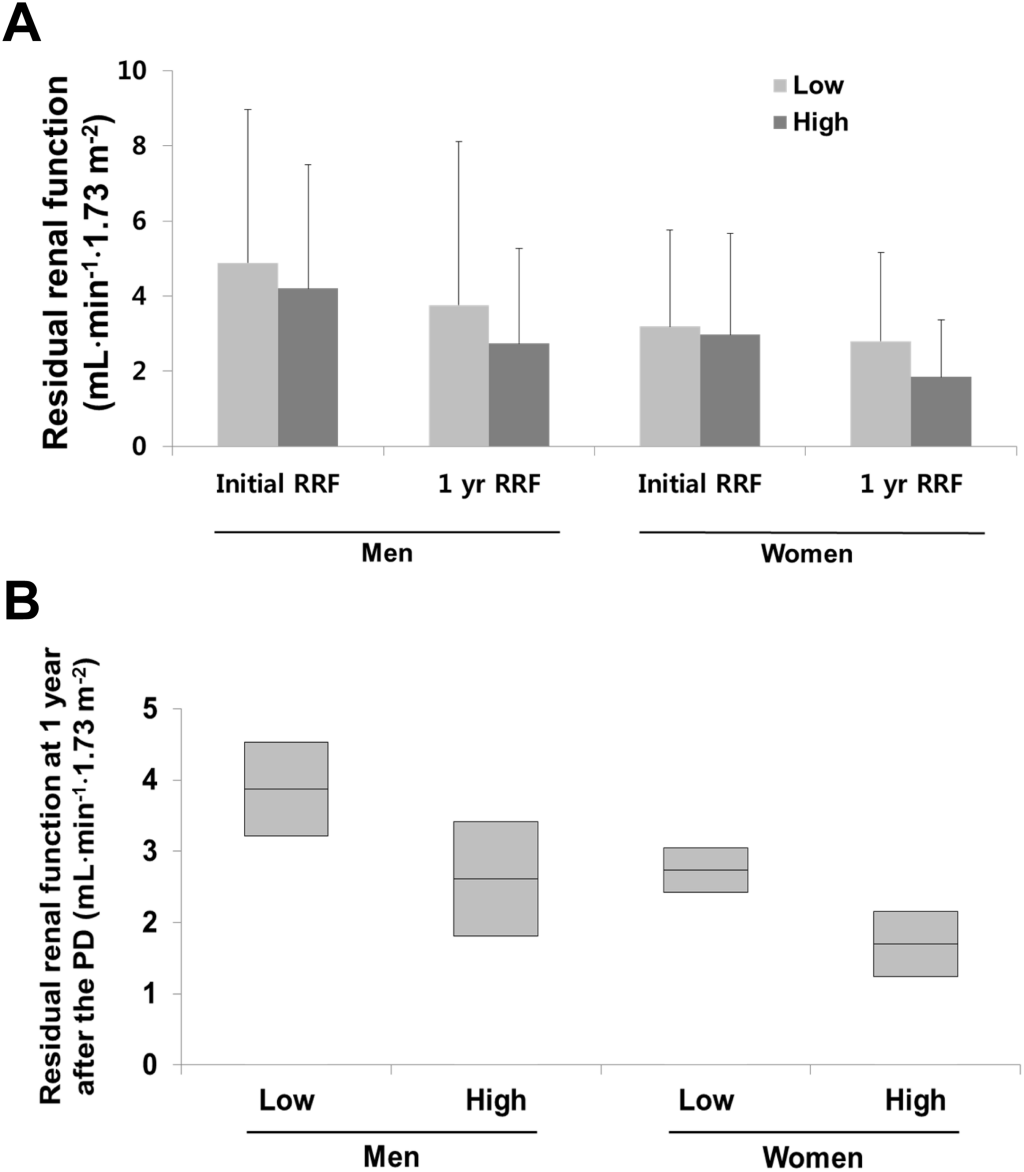
Changes in residual renal function (RRF) according to the initial edema index. A. Univariate analysis. B. Multivariate analysis. (A) RRF at peritoneal dialysis initiation and after a 1-year follow-up (the initial RRF in men was 4.88 ± 4.09 mL⋅min^-1^⋅1.73 m^-2^ and 4.21 ± 3.28 mL⋅min^-1^⋅1.73 m^-2^ in the Low and High groups respectively, *P* = 0.027; for women, 3.19 ± 2.57 mL⋅min^-1^⋅1.73 m^-2^ and 2.98 ± 2.70 mL⋅min^-1^⋅1.73 m^-2^ respectively, *P* = 0.001). (B) Multivariate analysis is adjusted for age, the Davies risk index, initial C-reactive protein level, and high transporter status. In the box plot, the horizontal line at the top, middle, and bottom of the boxes show the mean and 95% confidence interval (CIs) (in men, the means [95% CI] are 3.870 [3.211–4.529] and 2.611 [1.813–3.410] in the Low and high groups, respectively; *P* = 0.021; and in women, 2.734 [2.420–3.048] and 1.701 [1.243–2.160] in the Low and High groups, respectively; *P* < 0.001).

The respective mean initial RRF in the Low and High groups were 4.88 ± 4.09 mL·min^-1^·1.73 m^-2^ and 4.21 ± 3.28 mL·min^-1^·1.73 m^-2^ in men, and 3.19 ± 2.57 mL·min^-1^·1.73 m^-2^ and 2.98 ± 2.70 mL·min^-1^·1.73 m^-2^ in women (men: *P* = 0.108; women: *P* = 0.531; Fig 2A). There were no significant differences between groups of either sex. The respective mean RRF value at 1-year after PD initiation in the Low and High groups were 3.56 ± 4.35 mL·min^-1^·1.73 m^-2^ and 2.73 ± 2.53 mL·min^-1^·1.73 m^-2^ in men, and 2.80 ± 2.36 mL·min^-1^·1.73 m^-2^ and 1.85 ± 1.51 mL·min^-1^·1.73 m^-2^ in women. RRF at 1-year after PD initiation was higher in the Low group than in the High group (men: *P* = 0.027; women: *P* = 0.001). Multivariate analysis adjusted for age, the Davies risk index, initial CRP level, and high transporter status showed that RRF at 1-year after PD initiation was higher in the Low group than in the High group (Fig 2B).

In the FU cohort, the respective initial RRF in the Non-improvement and Other groups were 3.59 ± 2.47 mL·min^-1^·1.73 m^-2^ and 4.83 ± 4.41 mL·min^-1^·1.73 m^-2^ in men, and 3.36 ± 2.93 mL·min^-1^·1.73 m^-2^ and 2.71 ± 1.86 mL·min^-1^·1.73 m^-2^ in women (men: *P* = 0.088; women: *P* = 0.285). The respective RRF values at 1-year after PD initiation in the Non-improvement and Other groups were 2.42 ± 2.45 mL·min^-1^·1.73 m^-2^ and 3.74 ± 4.45 mL·min^-1^·1.73 m^-2^ in men (*P* = 0.018), and 1.93 ± 1.70 mL·min^-1^·1.73 m^-2^ and 2.37 ± 2.23 mL·min^-1^·1.73 m^-2^ in women (*P* = 0.246). There was a significant decrease in RRF in the Non-improvement group compared with the Other group in women (*P* = 0.013), but not in men (*P* = 0.917). Multivariate analysis showed a significant rapid decline in RRF at 1-year after PD initiation between the Non-improvement and Other groups in s women alone. In men, the means (95% CI) were –0.984 (–2.107, 0.139) and –1.239 (–1.849, –0.630) in the Non-improvement and Other groups, respectively (*P* = 0.701), while in women, values of –1.360 (–2.146, –0.573) and –0.370 (–0.705, –0.035) were observed in the Non-improvement and Other groups, respectively (*P* = 0.026).

### Survival analysis

**Fig 3:**
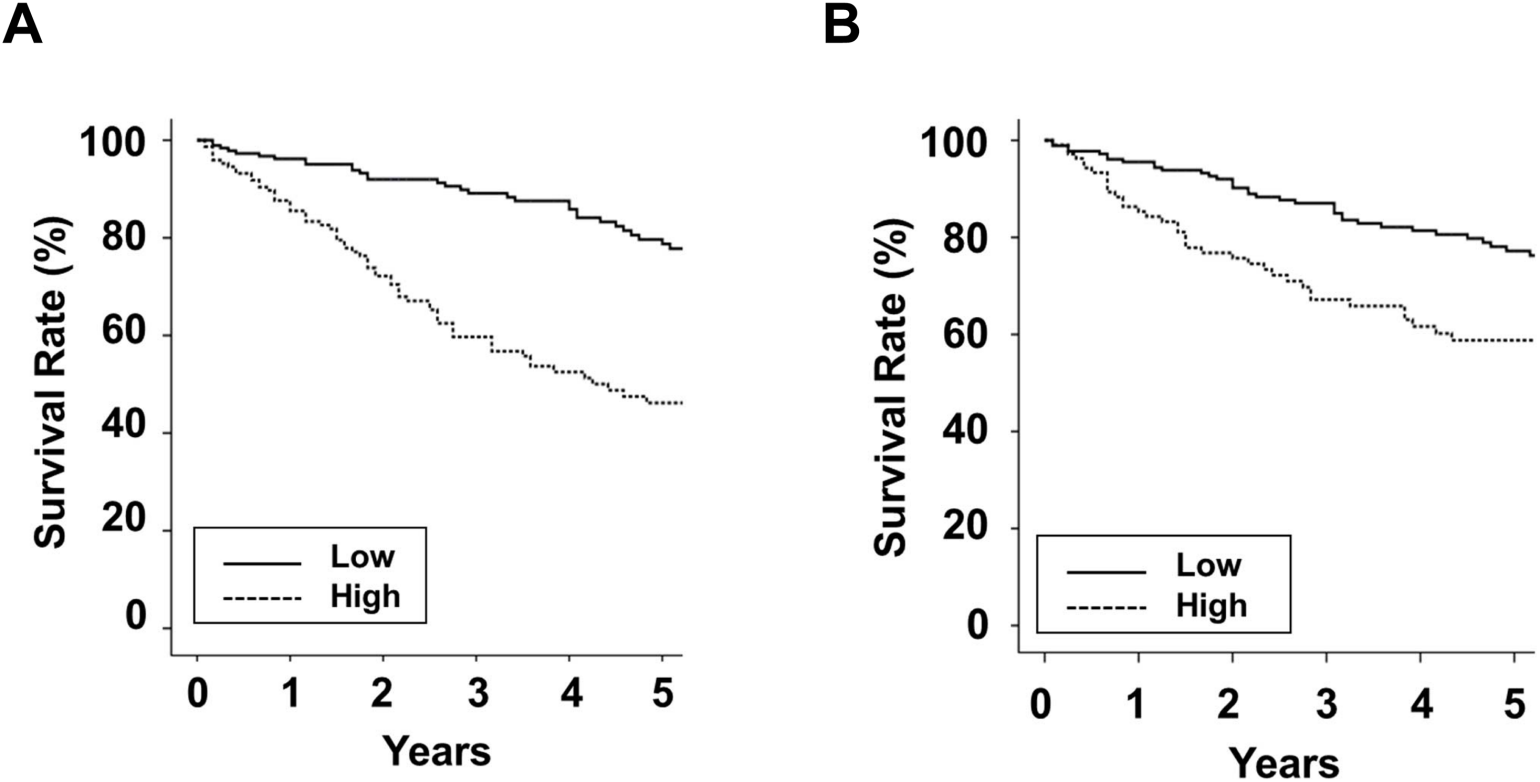
Kaplan-Meier survival curves according to the initial edema index group. (A) The survival rate in men (Low group: 96.2% at 1 year and 78.7% at 5 years; High group: 85.5% at 1 year and 46.2% at 5 years; *P* < 0.001). (B) The survival rate in women (Low group: 95.5% at 1 year and 77.2% at 5 years; High group: 85.3% at 1 year and 58.8% at 5 years; *P* < 0.001).

**Fig 4:**
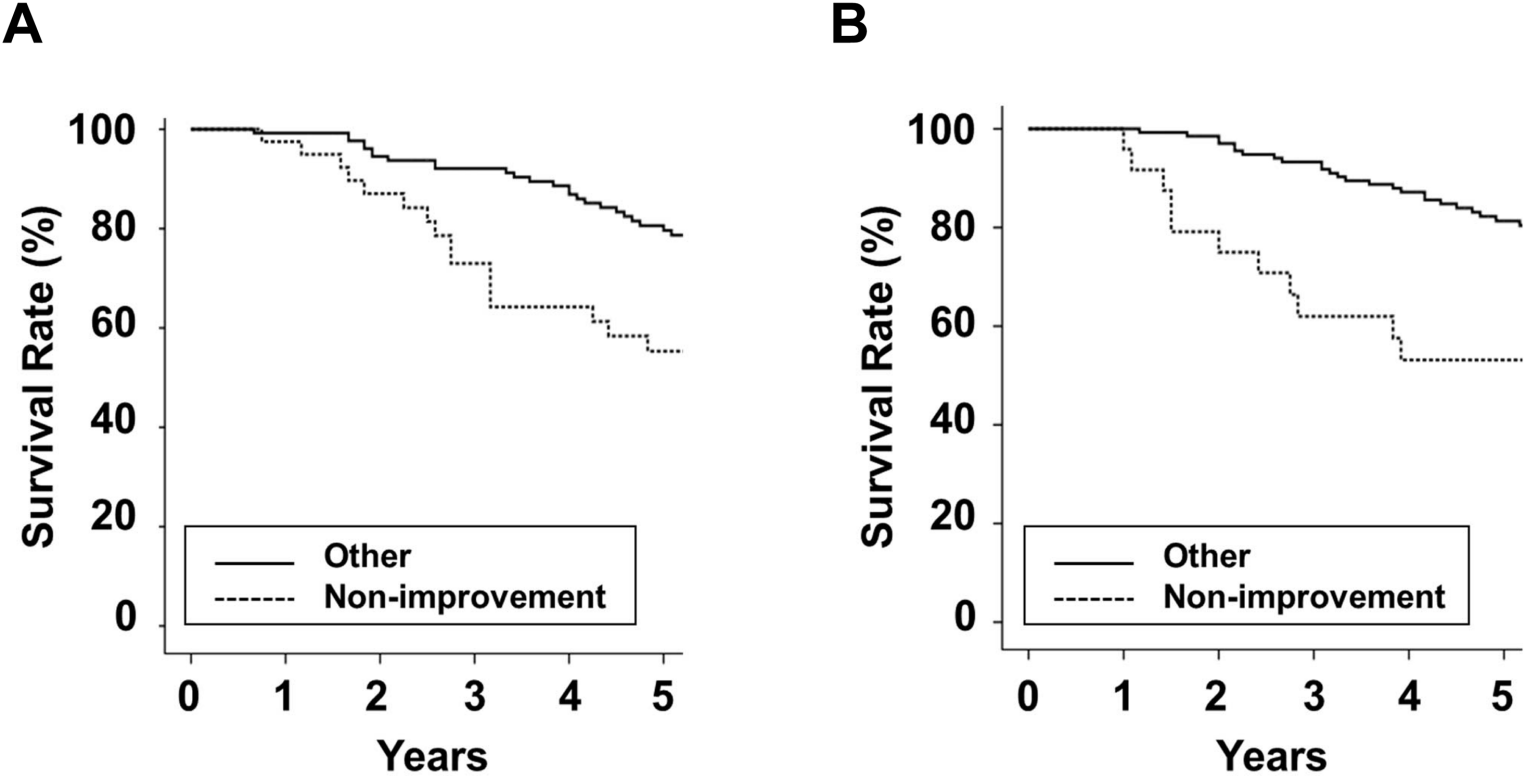
Kaplan-Meier survival curves according to changes in the edema index over a 1-year follow-up in peritoneal dialysis patients. (A) Survival rate in men (Other group: 79.6% at 5 years; Non-improvement group: 55.3% at 5 years; *P* < 0.001). (B) Survival rate in women (Other group: 81.3% at 5 years; Non-improvement group: 53.1% at 5 years; *P* < 0.001).

For the Total cohort, in men, the cumulative 5-year survival rates were 78.7% and 46.2% in the Low and High groups, respectively (Fig 3). In women, survival rates were 77.2% and 58.8% in the Low and High groups, respectively. Baseline high edema index was associated with mortality in PD patients. The leading cause of death was cardiovascular disease: 48 (38.7%) in men and 42 (42.4%) in women. In men, the proportion of death by cardiovascular disease was 15 (35.7%) and 33 (40.2%) in the Low and High groups, respectively and in women, 21 (39.6%) and 21 (45.7%) in the Low and High groups, respectively. For the FU cohort, the Non-improvement group was associated with a poor survival rate compared with the Other group for both sexes (Fig 4). In addition, multivariate cox regression analyses showed that the Low group or the Non-improvement group was associated with mortality in PD patients for both sexes (Table 3).

**Table 3:** Crude and adjusted hazard ratio for the risk of mortality according to the edema index groups.

Independent variables	Crude		Adjusted	
	Hazard ratio (95% CI)	*P*-value	Hazard ratio (95% CI)	*P*-value*
**Initial**				
Men				
Low	1.0	–	1.0	–
High	3.563 (2.462–5.157)	<0.001	2.703 (1.807–4.042)	<0.001
Women				
Low	1.0	–	1.0	–
High	1.989 (1.338–2.959)	0.001	1.755 (1.152–2.675)	0.009
**Follow-up**				
Men				
Other	1.0	–	1.0	–
Non-improvement	3.025 (1.843–4.966)	<0.001	2.477 (1.401–4.381)	<0.002
Women				
Other	1.0	–	1.0	–
Non-improvement	2.960 (1.641–5.340)	<0.001	2.616 (1.431–4.781)	0.002

*Multivariate analysis was adjusted for age, the Davies risk index, residual renal function, C-reactive protein level, and body mass index.

Abbreviation: CI, confidence interval.

## Discussion

The present study enrolled 631 incident PD patients and analyzed data at baseline and after the first year of PD initiation. The High group was defined as having a high edema index value, and this group was associated with mortality in PD patients in both univariate and multivariate analyses and a low RRF at 1-year after PD initiation. The Non-improvement group was also associated with mortality in PD survivors during the first year after PD initiation.

O’Lone et al. previously demonstrated an association between mortality and the edema index measured with bioimpedance spectroscopy in PD patients [20]. Their study reported a significant association between the two variables. However, the study enrolled both incident and prevalent PD patients, with only 225 incident PD patients. In addition, their survival data consisted of both incident and prevalent PD patients. Although patients underwent a transplant or were switched to hemodialysis during follow-up, data regarding these were not censored.

To our knowledge, this study is the first to evaluate the association between the edema index and mortality in Asian PD patients. The present study enrolled 631 incident PD patients. Although there was no significant difference in the edema index cutoff values by sex (men: 0.371; women: 0.372). The data were divided into men or women based on the difference in hydration status between the sexes. Additionally, patients who had undergone kidney transplantation or were switched to hemodialysis were analyzed as censored data. The survival data only included the PD duration. The data included the follow-up edema index at the first year after PD initiation. The results also indicated an association between mortality and the maintenance of a high edema index. Cardiovascular mortality in the Low group was greater than that in the High group, but no significant difference was observed between the groups.

RRF is an important survival factor in PD patients [21]. There have been conflicting findings regarding the association between volume status and RRF [22–26]. The association between the two variables is very complex, and volume status may be a cause or result for the loss of RRF. Several conflicting hypotheses have been proposed regarding the association between volume status and RRF. Some researchers have hypothesized that intravascular volume depletion by strict volume control can lead to a decrease in renal blood flow, which induces a loss in RRF through acute kidney injury [22–24,26]. Strict volume control may be harmful for preservation of RRF. Other researchers have suggested that overhydration is associated with hypertension and left ventricular hypertrophy, which can lead to a rapid decline in RRF [25]. They suggest that normalization of blood pressure control using strict volume control may play a role in preservation of RRF.

The present study showed a positive association between overhydration and loss of RRF. More specifically, initial RRF was not significantly different between the Low and High groups, but RRF at 1-year after PD initiation was higher in the Low group than in the High group. Multivariate analysis also showed a similar trend. In men, a difference in the initial RRF between the Non-improvement and Other groups was not observed, but RRF at 1-year after PD initiation was higher in the Other group than in the Non-improvement group. In women, the decreases in RRF during the 1-year follow-up in the Non-improvement and Other groups were –1.43 and –0.34, respectively. Multivariate analyses showed similar results in women. A woman will have a smaller volume of distribution of urea than a man. Therefore, a small volume change can induce greater effects in cardiovascular loading including blood pressure than in men. Although there was no significant difference in RRF at 1-year follow-up in the Non-improvement and Other groups in men, our results showed an inverse association between volume status and loss of RRF. Further investigations using serial follow-up data will be needed to identify the relationship between these two variables.

Many methods, such as dilution methods, chest radiography, inferior vena cava diameter, or BIA, have been introduced to evaluate the volume status. Dilution methods are known to be the reference methods for evaluating volume status. Although these are the most accurate methods, they are not useful in clinical practice. Cardiac diameter or pulmonary congestion using chest radiography is of value in monitoring changes over a longer time course, but these are not suitable for short-term monitoring [27]. Inferior vena cava diameter measurement using ultrasonography can help evaluate volume status, but currently available evidence is insufficient to prove efficacy in routine clinical practice [28]. In addition, the ultrasonography procedure is operator-dependent and can be affected by patient compliance [29]. The BIA has been the most extensively evaluated for predicting volume status and prognosis. Therefore, taking into consideration accuracy, ease of use, non-invasiveness, and low equipment cost, BIA may be recommended as a very useful method for evaluating volume status, when compared with other tools.

In the present study, the edema indexes were measured using BIA and were calculated using the ECF/TBF ratio. These measurements were different from the extracellular water (ECW)/total body water (TBW) ratio obtained from bioimpedance spectroscopy. Many researchers have used these two definitions as an interchangeable concept. A fluid is defined as water which has dissolved solutes, such as protein or mineral contents [30]. Therefore, normal values for the edema index vary between the two methods. Given fluid mainly consists of water, the edema index calculated by the ECF/TBF ratio is highly correlated with water contents measured by the dilutional method as a reference method. If a single method is used, the ECW/TBW ratio can be replaced with the ECF/TBF value.

In Korea, men partake in more occupational and social activities than women. Occupational and social activities may be associated with the predominance of APD. In addition, men had a higher baseline comorbidity index than women in our cohort. Although there was no clear cause for this phenomenon, it is known that men have more risk factors for cardiovascular diseases in the general population [31].

In the present study, there were significant differences in underlying comorbidities and baseline RRF between sexes. High comorbidities can induce rapid decline in RRF. On the other hand, participants with high comorbidities can initiate renal replacement therapy earlier than those with low comorbidities. This may be associated with high baseline RRF in participants with high Davies risk index. In our study, men had higher Davies risk indices than women and this was associated with higher RRF. Wright et al. analyzed the U.S. Renal Data System database and showed that a high baseline comorbidity index was associated with a high baseline RRF level [32]. Previous studies showed that the rate of decline in RRF is a more important prognostic factor than baseline RRF levels per se [33,34].

In the present study, RRF was higher in men than in women, conversely the Kt/V urea was higher in women than in men. Men have greater lean mass and less fat mass than women, and increased creatinine tubular excretion in dialysis can induce overestimation of creatinine clearance. In contrast, women are associated with a delay in the initiation of dialysis treatment due to reduced access to medical care, other economic factors, or patient attitudes [35,36]. Men also had a higher Davies risk index than women. These factors may be associated with greater RRF in men than in women. In addition, Watson et al. showed that the formula for men has a larger weight factor than the formula for women. This means that at the same BMI, a woman will have a smaller volume of distribution of urea than a man because Watson’s formula cannot perfectly correct for sex differences. A relatively smaller volume of distribution of urea may result in a relatively higher Kt/V in women [37]. Comorbidity conditions can also influence the Kt/V urea. However, in our study, there were no significant differences in Kt/V urea among the three Davies risk indices. Subgroup analyses divided by sex also showed similar results. These data indicate that differences in Kt/V urea between men and women were not associated with the Davies risk index.

Our data reveal that APD is associated with a high edema index and low serum albumin levels. Previous studies showed that APD was associated with a more rapid decline in RRF compared with CAPD [38–40]. Therefore, APD may result in a high edema index and low serum albumin level by a dilution-concentration effect. In addition, there was no significant difference in CRP levels between the Low and High groups for either sex. Fluid overload is associated with an increase in inflammatory markers such as CRP. Some studies have shown that the reduction of volume overload did not cause a decrease in serum CRP levels but was associated with an increase in serum albumin levels, a negative inflammatory marker [41–43].

In our study, UFF was defined only according to the PET results as established by the International Society for Peritoneal Dialysis guidelines [44]. The incidence of UFF was approximately 14.3%, and the Kt/V urea in men and women was 2.25 ± 0.76 and 2.45 ± 1.87, respectively. One multicenter cohort study using Koreans showed that the mean Kt/V urea value was 2.4 [45]. Although they did not report the incidence of UFF, the incidence of UFF in their study would likely be similar to that observed in our study. Considering the incidence of UFF, dialysis adequacy in our study was relatively high. Peritoneal inadequacy does not necessarily imply a failure in clinical management [19]. The definition of UFF in our study differs from that of previous reports. UFF has been mainly classified only in patients presenting with a clinical syndrome, but, in our study, UFF was defined only from results obtained from routine PET. The incidence of UFF was defined only based on PET results may be higher than in patients with UFF associated symptoms or signs. Although patients with UFF determined only by PET results had inadequate peritoneal membrane characteristics, none showed a clinical syndrome or low dialysis adequacy due to RRF. Previous studies have suggested that the prevalence of UFF is approximately 31–51% in PD patients [46–48]. However, only a few studies have evaluated the incidence of UFF, as most patients with a clinical syndrome defined only by peritoneal membrane characteristics would likely drop-out of a study during the early PD period. Patients with UFF only by PET results may be not evident at an early PD period due to a relatively high RRF.

In our study, APD utilization in high transporters was low. Generally, APD utilization in Korea is relatively low compared to that in the USA or Europe [49]. In Korea, patients who undergo APD pay additional fees for disposable lines in APD with a cost of approximately 77–111 USD per month. In addition, the life style of Koreans may be different from that of Americans or Europeans. Hence, high transporters in Korea prefer icodextrin use to APD.

The present study had a few limitations. First, this study was limited by its retrospective nature. In addition, this was a single-center study and it did not evaluate ethnic differences. The present study was an observational study, but also included incident PD patients. Therefore, dialysis vintage could be excluded from the limitations of the present study. Information regarding literacy was not collected in our study. The participants were all from the Asian population. Next, we used a single BIA measurement to evaluate the edema index. Although PD patients have a more stable volume status than hemodialysis patients, a time-averaged value using repeat measurements may be a more precise method for predicting mortality than a single value only. A prospective, multiethnic study, including additional parameters such as literacy or the averaged values using repeat measurements, is warranted to overcome these limitations.

In conclusion, a high edema index was found to be associated with mortality in incident PD patients at baseline and at follow-up. The present study suggested that the edema index may be used as a new marker for predicting mortality in PD patients. Hence, the edema index determined by BIA measurements should be used to closely monitor PD patients.

## Supporting Information

S1 Fig Study flow chart. (TIF)
